# Prenatal Stress Induces Long-Term Effects in Cell Turnover in the Hippocampus-Hypothalamus-Pituitary Axis in Adult Male Rats

**DOI:** 10.1371/journal.pone.0027549

**Published:** 2011-11-09

**Authors:** Eva Baquedano, Cristina García-Cáceres, Yolanda Diz-Chaves, Natalia Lagunas, Isabel Calmarza-Font, Iñigo Azcoitia, Luis M. Garcia-Segura, Jesús Argente, Julie A. Chowen, Laura M. Frago

**Affiliations:** 1 Department of Pediatrics, Universidad Autónoma de Madrid-Hospital Infantil Universitario Niño Jesús, Madrid, Spain; 2 CIBER Fisiopatología de Obesidad y Nutrición (CIBERobn), Instituto de Investigación Sanitaria Princesa, Instituto de Salud Carlos III, Madrid, Spain; 3 Laboratory of Neuroactive Steroids, Instituto Cajal, Consejo Superior de Investigaciones Científicas (CSIC), Madrid, Spain; 4 Department of Cellular Biology, School of Biology, Universidad Complutense de Madrid, Madrid, Spain; University of Córdoba, Spain

## Abstract

Subchronic gestational stress leads to permanent modifications in the hippocampus-hypothalamus-pituitary-adrenal axis of offspring probably due to the increase in circulating glucocorticoids known to affect prenatal programming. The aim of this study was to investigate whether cell turnover is affected in the hippocampus-hypothalamus-pituitary axis by subchronic prenatal stress and the intracellular mechanisms involved. Restraint stress was performed in pregnant rats during the last week of gestation (45 minutes; 3 times/day). Only male offspring were used for this study and were sacrificed at 6 months of age. In prenatally stressed adults a decrease in markers of cell death and proliferation was observed in the hippocampus, hypothalamus and pituitary. This was associated with an increase in insulin-like growth factor-I mRNA levels, phosphorylation of CREB and calpastatin levels and inhibition of calpain -2 and caspase -8 activation. Levels of the anti-apoptotic protein Bcl-2 were increased and levels of the pro-apoptotic factor p53 were reduced. In conclusion, prenatal restraint stress induces a long-term decrease in cell turnover in the hippocampus-hypothalamus-pituitary axis that might be at least partly mediated by an autocrine-paracrine IGF-I effect. These changes could condition the response of this axis to future physiological and pathophysiological situations.

## Introduction

Prenatal restraint stress in rats is a common experimental model of early stress known to have long-term behavioral and neurobiological consequences [1]–[4]. Subchronic stress during gestational life increases the levels of glucocorticoids prenatally, which is most likely involved in at least some of the adverse effects on metabolism, behavior and the neurological and immunological systems reported to occur in later life in both humans and rodents [2], [3], [5]–[8]. Prenatal stress modifies the plastic responses of the adult brain, including the circuitry of the hippocampus-hypothalamus-pituitary-adrenal axis (HHPA), that participate in the neuroendocrine control of feeding and metabolism in adult life [7], [9]. Indeed, glucocorticoids have a strong impact on fetal programming [1] with the brain being especially sensitive to this phenomenon. Depending on the magnitude, the duration and/or intensity of the stress, different effects on the central nervous system occur resulting in alterations in neurochemical systems, including activation of neurotransmission of serotonin and norepinephrine, among others [1], [10], changes in synaptic organization with atrophy of dendrites in the hippocampus [9] and reduction in the number of hippocampal synapses [11], [12], cerebral asymmetry and anomalies in the morphology of the brain [1], [13], as well as inhibition of cell death and neurogenesis [12]–[16]. These changes may thus condition the brain’s response to future physiological and pathophysiological situations.

Apoptosis is a genetically controlled active cell death process by which damaged or unnecessary cells are eliminated. This process is part of normal development and is induced by a great variety of physiological or pathological stimuli, both during development and in later life. Numerous proteins and transcription factors are implicated in this tightly regulated process [17], [18], [19], [20] with the most studied apoptotic pathways involving caspases, cystein-proteases that once activated by fragmentation, cleave protein substrates that finally lead to cell death [21], [22], [23], [24]. Calpains, which belong to a family of at least 14 members of calcium-dependent cysteine proteases, are also involved in apoptosis [25], [26]. These proteases are heterodimers composed of an 80-kDa catalytic subunit and a 28-kDa regulatory subunit that are associated with the endogenous calpain inhibitor, calpastatin [25]. Calpain substrates include cytoskeletal proteins [27], proteins involved in apoptosis such as Bax, p53, pro-caspases -9 and -3 and poly-ADP-ribose polymerase [28]–[31]. Increased expression levels of the endogenous calpain inhibitor calpastatin have been associated with reduced spinal cord injury and neuronal apoptosis [32], [33]. Calpains are implicated in a wide range of physiological functions including cell motility, differentiation, signal transduction, including cell survival pathways, cell cycle progression, regulation of gene expression and long-term potentiation [34], [35]. Insulin-like growth factor I (IGF-I) has neuroprotective actions and decreases calpain activation through activation of the Akt-CREB pathway resulting in anti-apoptotic actions [36].

Studies have shown that prenatal stress affects the fetal brain resulting in structural, emotional and neuroendocrine alterations postnatally [3], [4], [37], [38] and previous studies in our laboratory demonstrate that prenatal restraint stress alters cell turnover in the hypothalamus of adult male offspring [13]. In addition, the cellular composition of the pituitary can also be modified by early events with different cell populations being differentially susceptible to undergoing cell death in the adult [37], [39]–[41]. Thus, changes in its proliferative ability could modify its physiological activity. Hence, the aim of this study was to investigate if subchronic prenatal stress has an effect on cell death and proliferation in the hypothalamus-hippocampus-pituitary (HHP) axis of adult rats and to examine the mechanism involved. As long-term affectation of this axis could modify the animal’s response to future physiological challenges, with some of these modifications being possibly detrimental, understanding the mechanisms involved is important for the possible deterrence of adverse effects.

## Results

### Prenatal stress reduced basal cell death in the hippocampus, hypothalamus and pituitary in adult offspring

To quantify the cell death occurring in the hippocampus, hypothalamus and pituitary, a cell death detection ELISA was used. Prenatal stress reduced cell death in the hippocampus, hypothalamus and pituitary of the adult animal (Table 1).

**Table 1:** Relative levels of cell death and PCNA.

	Cell Death		PCNA	
	Control	PS	Control	PS
**Hippocampus**	100±11	52±6*	100±9	67±7*
**Hypothalamus**	100±7	60±4***	100±2	50±5***
**Pituitary**	100±8	41±1**	100±5	73±5**

Relative levels of cell death were assayed by ELISA and PCNA levels were measured by Western blotting in the hippocampus, hypothalamus and pituitary of control rats and prenatally stressed rats (PS). Data are expressed as means ± s.e.m. of three independent assays. Statistical significance by Student's t test:

*P<0.05,

**P<0.01 and

***P<0.001; n = 3–4/group.

### Prenatal stress reduced basal proliferation rate in the hippocampus, hypothalamus and pituitary of adult offspring

Determination of relative PCNA (proliferating cell nuclear antigen, a cofactor for DNA polymerase δ) levels by immunoblotting was used to evaluate cell proliferation at the time of sacrifice in the hippocampus, hypothalamus and pituitary. Prenatal stress decreased PCNA levels in all three areas studied (Table 1).

### Caspase and calpain pathways in the hippocampus, hypothalamus and pituitary

**Figure 1:**
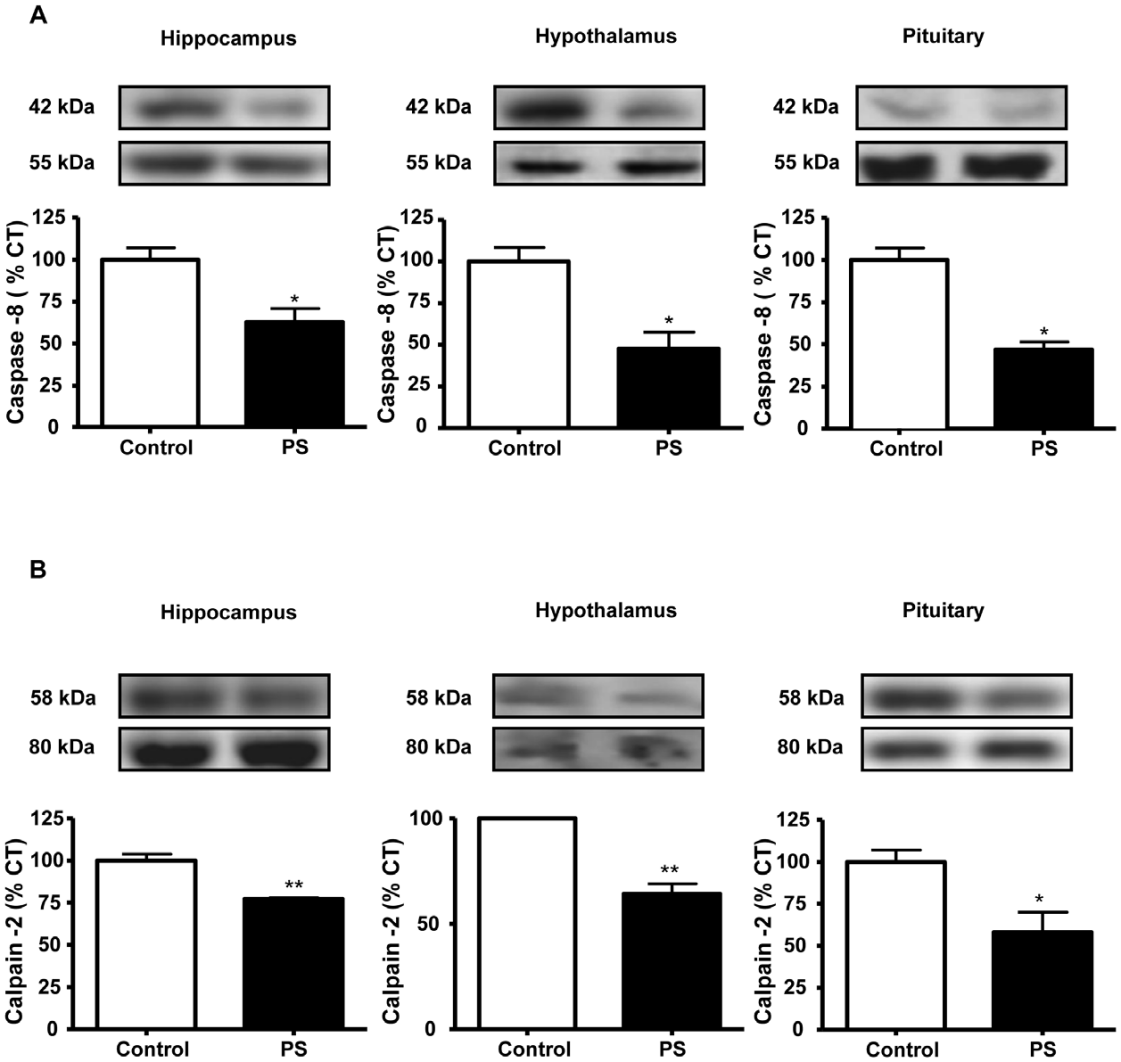
Prenatal stress reduces the fragmentation of caspase-8 and calpain-2. Immunoblots probed with antibodies towards caspase -8 (A) and calpain -2 (B) in the hippocampus, hypothalamus and pituitary of control rats and prenatally stressed rats (PS). The average of three independent assays is shown. Statistical significance by Student’s t test: * *P*<0.05 and ** *P*<0.01; n = 3–4/group.

To determine the mechanisms involved in the basal cell death in these tissues, we used immunoblotting to study the activation of the initiator caspases -8 and -9, of the extrinsic and intrinsic pathways of apoptosis, respectively. There were no changes in the activation (determined as percentage of fragmentation of the proform) of caspase-9 in response to prenatal stress (data not shown). However, in rats subjected to prenatal stress there were decreased levels of caspase-8 fragmentation in the three areas studied (hippocampus: 63% of control values, hypothalamus: 47% of control values, and pituitary: 46% of control values; Fig. 1A).

Another group of proteases involved in apoptosis is the calpain family. We estimated calpain-2 activation by Western blotting determining the relative fragmentation of the 80-kDa catalytic subunit into the 58-kDa active form. Prenatal stress reduced the fragmentation of calpain-2 in the hippocampus (77% of control values), hypothalamus (64% of control values) and pituitary (58% of control values) (Fig. 1B).

### Calpastatin levels in the hippocampus, hypothalamus and pituitary

**Figure 2:**
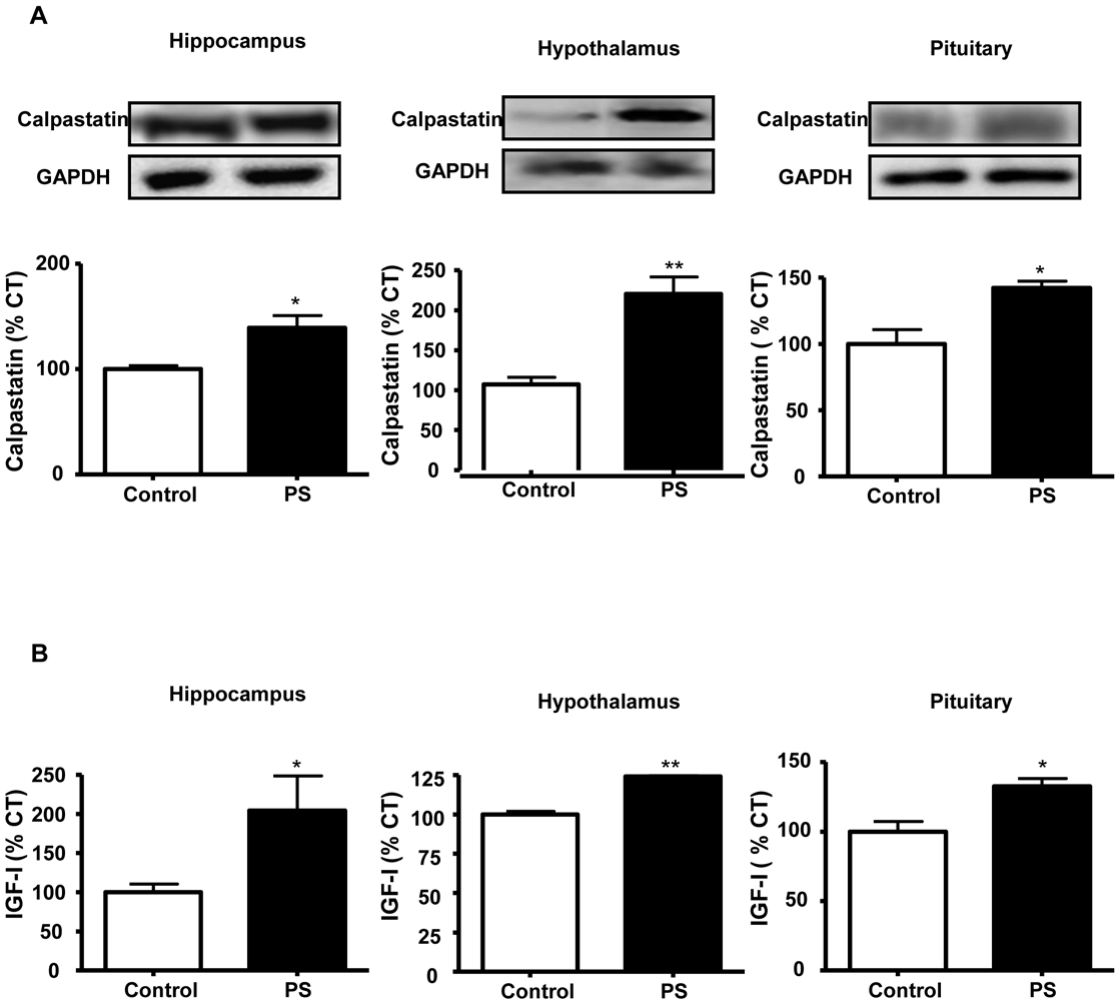
Prenatal stress increases calpastatin and IGF-I mRNA levels. (A) Immunoblots probed with antibodies towards calpastatin in the hippocampus, hypothalamus and pituitary of control rats and prenatally stressed rats (PS); (B) Relative mRNA levels of IGF-I in the hippocampus, hypothalamus and pituitary of control and PS rats. The average of three independent assays is shown. Statistical significance by Student’s t test: * *P*<0.05 and ** *P*<0.01; n = 3–4/group.

The endogenous calpain inhibitor, calpastatin, was measured by immunoblotting. Prenatal stresses increased the levels of calpastatin in the hippocampus (140% of control values), hypothalamus (220% of control values) and pituitary (143% of control values; Fig. 2A).

### IGF-I levels

An increase in IGF-I mRNA levels was found in prenatally stressed rats in the three areas studied (hippocampus: 204%, hypothalamus: 125% and pituitary: 132% of control values; Fig. 2B).

Prenatal stress did not modify serum levels of IGF-I. Mean IGF-I concentration in control rats was 1257±14 ng/ml and 1180±38 ng/ml in prenatally stress rats.

### Regulation of apoptotic pathways

#### 1. Bcl-2 family

**Figure 3:**
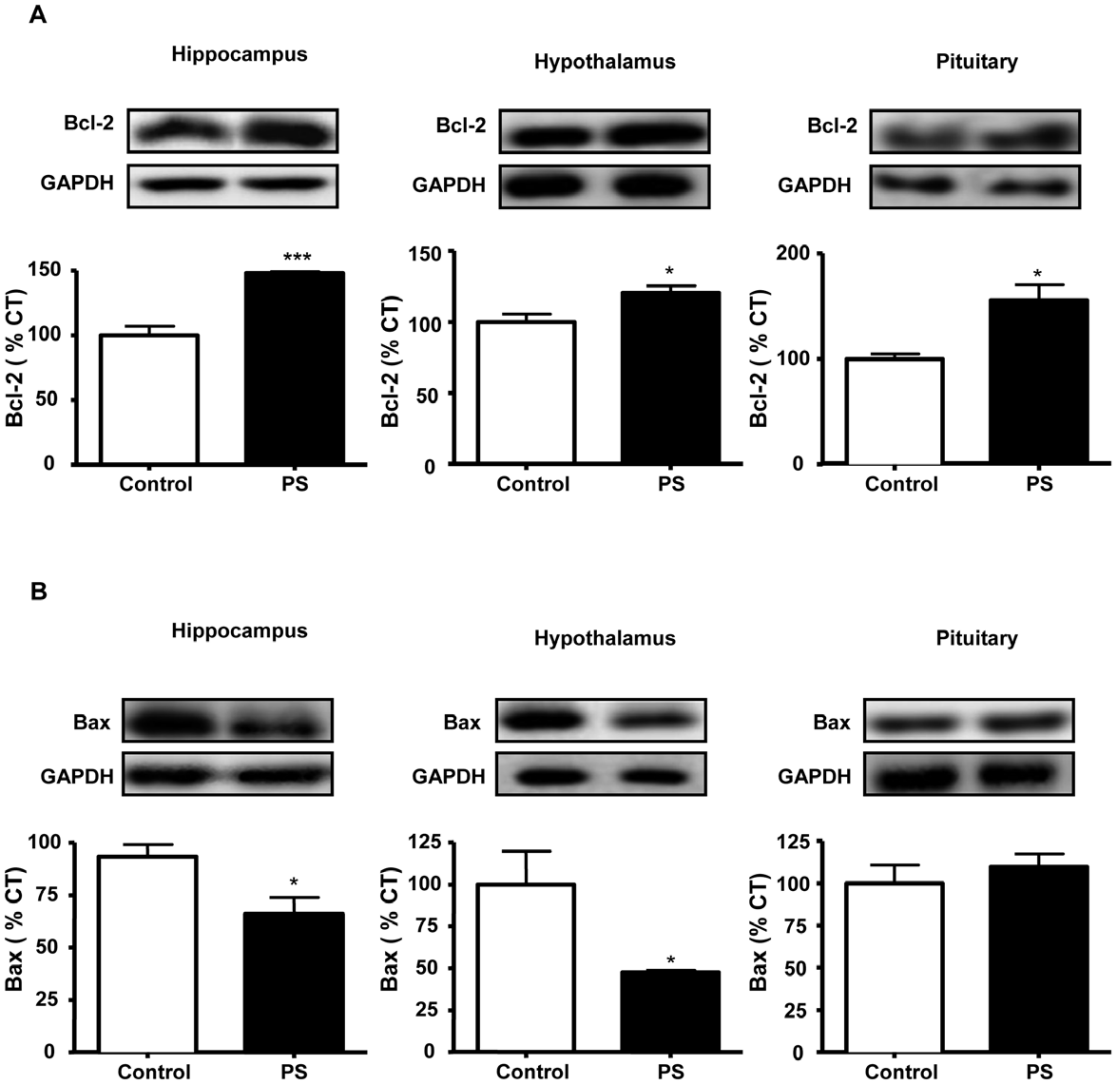
Effects of prenatal stress on the levels of Bcl-2 and Bax. Immunoblots probed with antibodies towards Bcl-2 (A) and Bax (B) in the hippocampus, hypothalamus and pituitary of control rats and prenatally stressed rats (PS). Statistical significance by Student’s t test: * *P*<0.05; ** *P*<0.01 and *** *P*<0.001; n = 3-4/group.

Levels of pro- and anti-apoptotic members of Bcl-2 family were measured by immunoblotting. Prenatal stress increased the levels of the anti-apoptotic protein Bcl-2 in the hippocampus (148% of control values), hypothalamus (121% of control values) and pituitary (156% of control values; Fig. 3A).

In the hippocampus and hypothalamus a decrease in Bax levels was observed in response to prenatal stress (hippocampus: 66% of control values; hypothalamus: 47% of control values). The levels of the pro-apoptotic protein Bax did not change in the pituitary (Fig. 3B).

#### 2. p53

**Figure 4:**
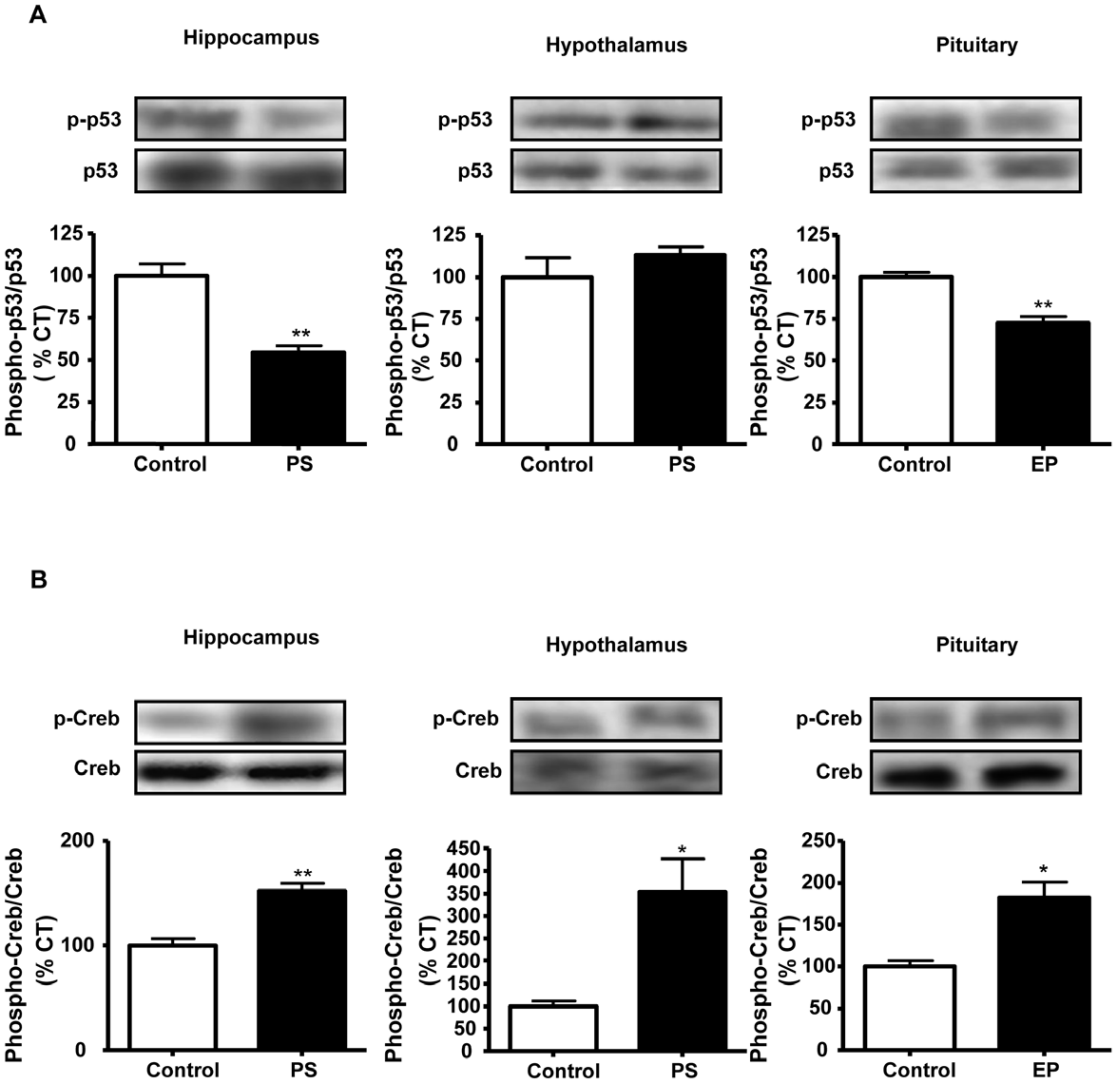
Effects of prenatal stress on the levels of p-53 and CREB. Immunoblots probed with antibodies towards phosphorylated (p)-p53 (A) and p-CREB (B) in the hippocampus, hypothalamus and pituitary of control rats and prenatally stressed rats (PS). The average of three independent assays is shown Statistical significance by Student’s t test: * *P*<0.05 and ** *P*<0.01; n = 3–4/group.

We studied p53, an important protein involved in apoptosis regulation as its main function is to repair damaged DNA and in case of major damage it induces apoptosis. We used immunoblotting to measure the level of phosphorylation of p53 (p-p53), which activates this protein, and observed that p-p53 levels were decreased in the hippocampus (54% of control values) and pituitary (72% of control values) of prenatally stressed rats, with no changes in the hypothalamus (Fig. 4A).

#### 3. CREB

We analyzed the activation of CREB since IGF-I and calpastatin induce the phosphorylation of this factor. Prenatal stress increased the levels of p-CREB in the three areas studied (hippocampus: 152% of control values; hypothalamus: 353% of control values and pituitary: 182% of control values; Fig. 4B).

## Discussion

The exposition to high levels of stress hormones during development can cause long-term effects [2], [42], [43]. Changes in the microenvironment and in cell survival in the hippocampus have been reported in response to maternal stress [44], [45]. Here we show that prenatal stress decreases proliferation markers in the hypothalamus of adult male rats, in agreement with our previous report [13], but also that a similar phenomenon occurs in the hippocampus and pituitary. Although previous studies have shown increased cell death in neurons of the hypothalamic paraventricular nucleus in fetal rats [46], studies in adult rats show a reduction in hippocampus cell proliferation in response to prenatal restraint stress and report that it is not accompanied by an increase in pyknosis [5], [47]. This is in accordance with the data presented here as we found that prenatal stress not only reduced the rate of cell proliferation, but also inhibited cell death in the adult hippocampus. This reduction in cell death also occurred in the hypothalamus and the pituitary. The long-term effect of prenatal stress on cell death and proliferation reported here could be related to the “glucocorticoid cascade” hypothesis, which proposes that stressful experiences are responsible for alterations in the structure and function of the hippocampal formation through an excessive release of corticosterone [48], [49]. However, this effect would most likely be due only to prenatal exposition to corticosterone, as the levels of corticosterone at sacrifice were similar in all rats [50] and there was no effect on adrenal gland weight, as reported here. Taken together, these data suggest a slowing of the cell cycle in the HHP axis of prenatally stressed rats. Prenatal stress induces apoptosis in different areas of the fetal or neonatal brain, including neurons of the hypothalamic paraventricular nucleus [46]. This suggests that stress may have a greater effect on immature cells, which may be more susceptible to cell death prior to their establishment of firm connections [51], [52].

To study the intracellular mechanisms involved in the reduction of cell death, apoptotic pathways were analyzed. Fragmentation of caspase-8 was reduced in the HHP axis in prenatally stressed rats. Calpains, a family of Ca^2+^-dependent cystein proteases involved in neuronal apoptotic processes after different injuries [53], [54] are known to act as regulator of caspases [55] and many calpain substrates are similar to, or their functions overlap with, those of caspases [42], [56]. Prenatal stress inhibited cleavage of calpain-2 in the HHP axis of adult offspring. Calpain levels are regulated by an endogenous inhibitor, calpastatin that exerts neuroprotective actions [32], [57]. We observed an increase of calpastatin levels in the three areas studied in prenatally stressed rats. Thus, this up-regulation of calpastatin could help to explain the decrease in calpain-2 and apoptosis.

An important regulatory step in apoptosis occurs at mitochondrial membranes involving the members of the Bcl-2 family of proteins. The levels of the anti-apoptotic protein Bcl-2 were increased in the HHP axis in the prenatally stressed rats. In contrast, the levels of the pro-apoptotic protein Bax were reduced in the hippocampus and hypothalamus of prenatally stressed rats. Hence, prenatal stress up-regulated Bcl-2 and down-regulated Bax, resulting in an anti-apoptotic balance. This rise in Bcl-2 possibly is involved in the inhibition of caspase-8 activation [58]. The increased expression of Bcl-2 also provides a mechanism to inhibit the opening of ionic channels resulting in an accumulation of calcium, as a consequence the cell is more resistant to calcium induced injuries [59], [60]. The prolonged exposure to calcium would lead to an increase in calpastatin expression, which could be a cellular mechanism of protection against alterations of the intracellular calcium homeostasis [61].

The tumor suppressor protein p53 is activated in response to cell stress leading to cell cycle arrest and apoptotic cell death. p53-induced cell death leads to the activation of caspases by release of apoptogenic factors from mitochondria with this process being regulated by the Bcl-2 family of proteins [18], [62], [63]. Our data show that phosphorylation of p53 is decreased in the hippocampus and in the pituitary with no effect in the hypothalamus in rats subjected to prenatal stress. The observed inhibition of p53 could be the result of the increase in Bcl-2 in those areas.

The transcription factor CREB is proposed to be involved in protecting the brain after a stressful situation [64], [65]. In addition, the calpastatin promoter sequence contains single cAMP-response element [36], [66], [67]. Prenatal stress increased CREB phosphorylation in the HHP axis, which could explain the increase in calpastatin levels and the following inhibition of calpain and caspase activation. Moreover, CREB regulates the expression of Bcl-2, Bax, and p53 in different cells [36], [68]. As CREB is involved in IGF-I induced neuron survival [36], [65] and is involved in metabolic homeostasis and growth during development [68], [69], IGF-I could be involved in the changes observed here. In support of this, we found that prenatal stress increased IGF-I mRNA levels in the three areas studied. Circulating levels of IGF-I in blood were similar in both experimental groups. These data suggest that IGF-I could be acting in an autocrine-paracrine manner regulating the calpastatin-calpain system via CREB to inhibit cell death.

**Figure 5:**
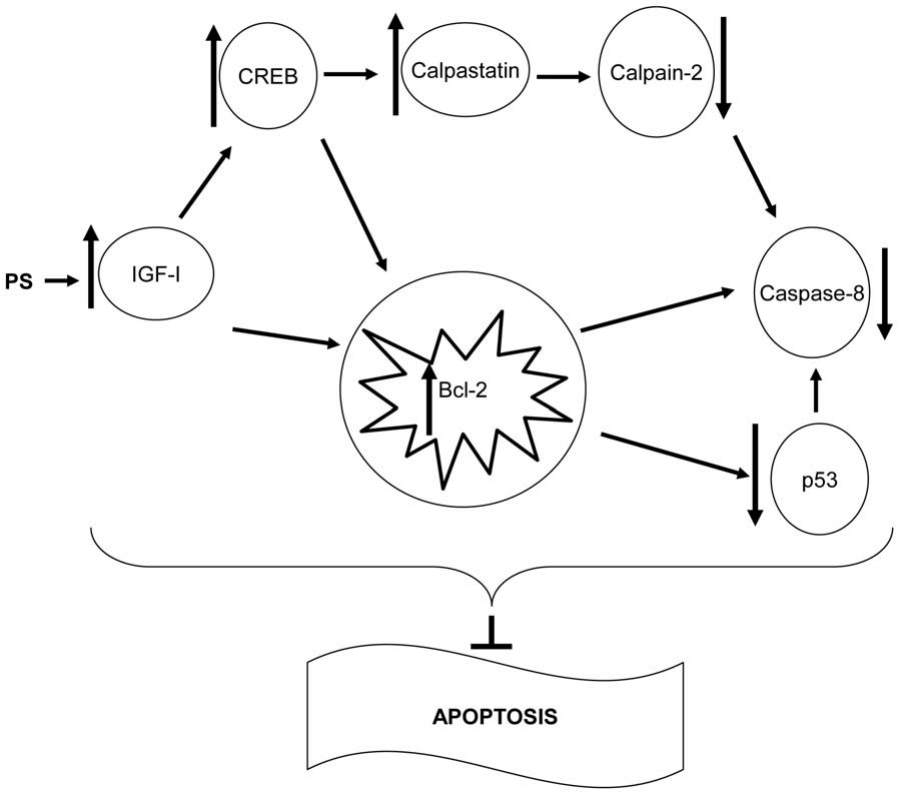
Diagram representing the mechanism proposed for prenatal stress inhibition of cell death . Prenatal stress would induce an increase in local IGF-I levels that would induce an increase in calpastatin levels via CREB that would in turn inhibit calpain -2. Furthermore, IGF-I would stimulate Bcl-2 leading to decreased levels of p-p53 and finally to a less activation of the extrinsic apoptosis pathway.

In summary, our data suggest that prenatal stress induces a long-term slowing or deceleration in the cell death and proliferation rate in the HHP axis. The increase in local IGF-I levels could be involved in the increase in calpastatin levels via CREB that would in turn, inhibit calpain-2 resulting in decreased activation of the extrinsic apoptosis pathway (Fig. 5). Exposure of the developing brain to stress is known to increase the individual’s vulnerability to mental disorders [3]. In addition, not only can the long-term stress response be affected by this slowing of cell-turnover in the HHP axis, but other endocrine functions could be modulated in the adult rat due to the affection of the hypothalamus and pituitary. This slowing of cell turnover could result in a reduction in their sensitivity to both physiological and pathophysiological changes with, for example, their susceptibility to future metabolic challenges possibly being modified. It is known that a high fat diet induces obesity [70] and this is associated with an inhibition of neurogenesis [71], as well as the induction ofapoptosis of hypothalamic neurons [72]. Hence, it is possible that the slowing down of cell turnover of the HHP axis could be involved in the vulnerability to metabolic imbalances or other diseases.

## Materials and Methods

### Materials

Electrophoresis reagents were from BioRad Laboratories (Hercules, CA, USA) and the rest of chemicals and reagents were purchased from Sigma Chemical Co. (St. Louis, MO, USA) or Merck (Barcelona, Spain) unless otherwise indicated.

### Animals

All studies were approved by the ethics committee of the Universidad Autónoma de Madrid (BFU2008-02950, Ministerio de Ciencia e Innovación) and complied with the Royal Decree 1201/2005 (Boletín Oficial del Estado, BOE no. 252) pertaining to the protection of experimental animals and with the European Communities Council Directive (86/609/EEC).

Young adult Wistar rats (10–14 weeks of age) from Harlan Laboratories (Indianapolis, IN, USA) were housed individually and maintained in a ventilated room at a constant temperature (22°C) and humidity (50%) on a 12 h light/dark cycle and allowed free access to rat chow and tap water and were allowed to acclimate to the new environment for one week. Afterwards, one female was placed with a male for four days. During these days, vaginal smears were carried out to determine the first day of gestation and dams were housed individually to proceed with the experiment.

Prenatal restraint stress in rats is a validated model of early stress resulting in permanent behavioral and neurobiological consequences [3], [4]. Restraint stress was performed daily in pregnant rats during the last week of gestation (gestation days 14–21) by placing them in transparent plastic cylinders (7 cm in diameter, 19 cm long) with bright light exposure for 45 minutes, three times a day at 0900, 1200 and 1700 hours during the dark phase of the animals, as previously described [73]. Pregnant rats from the control group remained undisturbed in their home cage. At birth, pups remained with the dam with no handling of either the pups or the mothers until postnatal day 21 (P21) at which time they were weaned. Although there were no differences in mean litter size between controls and stressed dams, only litters consisting of 9–14 pups were employed in the study to avoid changes in body weight or food intake due to litter size. At P21, pups were distributed (four/cage) according to their origin from control or stressed dams, with males and females being housed separately. Ten animals per experimental group were employed with these animals coming from 3 different litters to reduce a possible litter effect. All rats were killed by decapitation. Trunk blood was collected, allowed to clot and then centrifuged at 3000 rpm. Serum was separated and stored at −70°C until processed. The hippocampi, hypothalami and pituitaries were isolated and stored at -70°C until processed. The adrenal glands were removed and weighed with no significant differences being found (mean adrenal gland weight in control rats was 59±10 mg and 52±3 mg in prenatally stressed rats). Only male rats (n = 9–10/group) were studied.

### Cell death detection ELISA

This assay was carried out according to the manufacturer’s instructions (Roche Diagnostics, Mannheim, Germany). Briefly, tissue was homogenized in incubation buffer and microtiter plates were coated with anti-histone antibody. The samples were added (in duplicate) and incubated (90 min, RT). The wells were washed and incubated with anti-DNA-peroxidase (90 min, RT). After washing, substrate solution was added until the color developed adequately (approximately 15 min). The samples were measured at 405 nm on an automatic microplate analyzer (Tecan Infinite M200, Grödig, Austria). Background measurements at 490 nm were made and this value subtracted from the mean value of each sample.

### Tissue homogenization and protein quantification

Hypothalami, hippocampi and pituitaries were homogenized on ice in 200 µl of RIPA buffer with an EDTA-free protease inhibitor cocktail (Roche Diagnostics). After homogenization, samples were centrifuged at 12000 g for 20 min at 4°C. Supernatants were transferred to new tubes and protein concentration was measured using the BioRad Protein Assay (BioRad).

### Immunoblotting

In each assay the same amount of protein was loaded in all wells (30–60 µg depending on the protein to be detected) and resolved using 8–15% SDS-PAGE and then transferred onto PVDF membranes (BioRad). Filters were blocked with Tris-buffered saline containing 0.1% (v/v) Tween 20 and 5% (w/v) BSA or non fat milk and incubated overnight at 4°C with the primary antibody at a dilution of 1:1000 in blocking buffer. Primary antibodies included those for p53 and caspase -8 from Neomarkers (Fremont, CA, USA), caspase -9 from Medical Biological Laboratories (Woburn, MA, USA), Bcl-2 (B-cell lymphoma 2) and Bax (BCL2-associated X) from Thermo Scientific (Cheshire, UK), phospho-p53 (p-p53), phospho-CREB (cAMP response element-binding; p-CREB) and CREB from Cell Signaling Technology (Beverly, MA, USA), calpain -2 from Chemicon International (Temecula, CA, USA), calpastatin from Santa Cruz Biotechnology (Santa Cruz, CA, USA), proliferating cell nuclear antigen (PCNA) from Signet (Dedham, MA, USA). Filters were washed and incubated with the corresponding secondary antibodies conjugated with peroxidase at a dilution of 1:2000 (Pierce, Rockford, IL, USA). Bound peroxidase activity was visualized by chemiluminiscence (PerkinElmer life Science, Boston, MA, USA) and quantified by densitometry using a Kodak Gel Logic 1500 Image Analysis system and Molecular Imaging Software, version 4.0 (Rochester, NY, USA). All blots were re-blotted with glyceraldehyde-3-phosphate dehydrogenase (GAPDH; AnaSpec, San Jose, CA, USA) to normalize each sample for gel-loading variability. Phosphorylated proteins were normalized to non-phosphorylated levels and fragmented to non-fragmented. All data were normalized to control values on each membrane.

### Immunoenzymometric assay (IEMA) for determination of insulin-like growth factor I (IGF-I)

The quantitative determination of serum IGF-I was performed with the OCTEIA immunoenzymometric assay from IDS, Immunodiagnostic Systems Limited (Boldon, Tyne & Wear, UK). The method incorporates a sample treatment to avoid interference from binding proteins. The method was performed according to the manufacturer’s instructions. Briefly, serum samples were incubated with a reagent to inactivate binding proteins (10 min, RT) and then diluted for assay. In the OCTEIA rat/mouse IGF-I kit, a purified monoclonal anti-rat IGF-I is coated onto the inner surface of polystyrene microtiter wells (the solid phase or capture antibody). The pretreated, diluted samples were then incubated with biotinylated polyclonal rabbit anti-rat IGF-I, in antibody-coated wells for 2 h, RT on a shaking platform. The wells were washed and horseradish peroxidase labeled avidin, which binds to the biotin complex, was added (30 min, RT). After washing, a single component chromogenic substrate (a formulation of tetra-methyl-benzidine) was added to develop color (30 min, RT). The reaction was stopped and the absorbance read (450 nm; reference 650 nm) in a microtiter plate reader, with color intensity being directly proportional to the amount of rat IGF-I present in the sample. This assay has a sensitivity limit of 63 ng/ml. The intra- and interassay coefficients of variation were 4.3% and 6.3%, respectively.

### RNA purification

Total RNA was purified following the instructions of TriReagent (Invitrogen, Carlsbad, CA, USA). Briefly, 100 mg of hippocampus, hypothalamus or the whole pituitary were homogenized in 1 ml of TriReagent and incubated 5 min at RT to dissociate nucleoprotein complexes. Chloroform (0.2 ml) was added and samples were vortexed, incubated 15 min at RT and then centrifuged at 12000 g for 15 min at 4°C. The aqueous phase was transferred to new tubes and isopropanol (0.5 ml) was added to precipitate RNA. Samples were incubated 10 min at RT and then centrifuged at 12000 g for 10 min at 4°C. Pellets were washed in 75% ethanol (1 ml), centrifuged at 7500 g for 5 min at 4°C, and dissolved in RNase-free water. Absorbance at 260 nm was measured to determine concentrations.

### Real Time PCR (polymerase chain reaction)

cDNA was synthesized from 2 µg of total RNA by using the high capacity cDNA reverse transcription kit (Applied Biosystems, Foster City, CA, USA). Quantitative real-time PCR was performed by using assay-on-demand kits (Applied Biosystems) for IGF-I (Rn 99999087_m1) and TaqMan Universal PCR Master Mix (Applied Biosystems) were used according to the manufacturer’s protocol in an ABI PRISM 7000 Sequence Detection System (Applied Biosystems). Values were normalized to the housekeeping gene GAPDH (Rn 99999916_s1). According to manufacturer’s guidelines, the ΔΔC_T_ method was used to determine relative expression levels. Statistics were performed using ΔΔC_T_ values.

### Statistical analysis

Statistics were performed using the statistical program GraphPad Prism 4.0. Data are presented as means ± S.E.M. Student's t test were performed. The values were considered statistically significant when the *P* was < 0.05.
